# A Scoping Review of Populist Radical Right Parties’ Influence on Welfare Policy and its Implications for Population Health in Europe

**DOI:** 10.34172/ijhpm.2020.48

**Published:** 2020-04-08

**Authors:** Chiara Rinaldi, Marleen P.M. Bekker

**Affiliations:** Health and Society Group, Wageningen University & Research, Wageningen, The Netherlands.

**Keywords:** Populist Radical Right, Welfare Policy, Europe

## Abstract

**Background:** In light of worrying public health developments such as declining life expectancy gains and increasing health inequalities, there is a heightened interest in the relationship between politics and health. This scoping review explores the possible welfare policy consequences of populist radical right (PRR) parties in Europe and the implications for population health. The aim is to map the available empirical evidence regarding the influence of PRR parties on welfare policy reforms and to understand how this relationship is mediated by political system characteristics in different countries.

**Methods and Analysis:** A scoping review of peer-reviewed empirical literature addressing the relationship between PRR parties, political systems and welfare policy in Europe was performed using the methodology by the Joanna Briggs Institute. Data was charted on main study characteristics, concepts and relevant results, after which a qualitative content analysis was performed. The data was categorised according to the political system characteristics: constitution, political economy, interest representation and partisanship. Five expert interviews were conducted for validation purposes. Early evidence from 15 peer-reviewed articles suggests that exclusionary welfare chauvinistic positions of PRR parties are likely to have negative effects on the access to welfare provisions and health of vulnerable population groups. Differences in implementation of welfare chauvinistic policy reforms are partly explained by mediation of the constitutional order (judicial institutions at national and European Union [EU] level), political economy (healthcare system funding and European single market) and partisanship (vote-seeking strategies by PRR and mainstream parties). No clear evidence was found regarding the influence of interest representation on welfare chauvinistic policies.

**Discussion:** While early evidence suggests that the welfare chauvinistic ideology of PRR parties is harmful for public health, the possible mediating role of political system characteristics on PRR welfare policy influence offers risk and protective factors explaining why the PRR ideology plays out differently across Europe.

## Background

In recent years, a worrying reversal of public health trends can be noted in a number of European countries, including declines in life expectancy gains, increasing health inequalities and disinvestment in public health due to austerity measures.^1,2^ In explaining current health challenges, public health scholars are increasingly interested in the political determinants of health.^3^ Politics is the collective selection and legitimation of public policies for the achievement of goals and resolution of problems.^4^ In other words, ‘Who gets to decide what, when and how,’ which to a large extent determines how public policies affect health.^5^

The rise of populist radical right (PRR) parties in Europe has brought public health scholars to raise attention for the possible health consequences of their influence on public policies.^6^ The popularity of PRR parties is usually attributed to economic inequalities in society and backlash against immigration, which have led to increased dissatisfaction with the current political system.^7^ Political systems are here defined as consisting of (1) a constitution (including separation of powers and the role of the judiciary), (2) political economy (trade policies, fiscal policies, labour markets and socio-economic policies), (3) interest representation (activities carried out to influence policy formulation and decision-making processes) and (4) partisanship (an individual’s preference for the victory of one party over another).^3^

So, what is the PRR? Populism is a ‘thin-centred ideology’ that creates a hypothetical division between ‘the pure people’ and ‘the corrupt elite,’^8^ in which the elite usually includes mainstream political parties, the media, the upper classes, intellectuals and the European Union (EU). PRR parties are furthermore characterized by nativism (believing in an ethnically united people with a territory) and authoritarianism (believing in the value of obeying and valuing authority, granted that it is their own).^8^ Examples of PRR parties in Europe are the Freedom Party of Austria (FPÖ), Swiss People’s Party (SVP), the League (LN, Italy) and former conservative parties Fidesz (Hungary) and Law and Justice (PiS, Poland). While Donald Trump and Boris Johnson are often considered PRR leaders, the Republican Party (US) and the Conservatives (UK) are not PRR parties. According to De Cleen,^9^ the combination of populism and nativism that characterises PRR parties could form a threat to universal access to healthcare, and negatively affect the health of vulnerable groups. Welfare chauvinism is believed to play a central role in this. The term welfare chauvinism refers to increasing welfare benefits for the hypothetical ‘in-group’ while restricting access and eligibility for the ‘out-group.’^10^ In addition, the PRR’s scepticism towards the elite is suggested to extend to their beliefs about medical expertise. An example of this are the anti-intellectual messages against vaccination that are spread by some PRR party leaders.^11^

Despite the increasing popularity of PRR parties and their participation in government in several European countries,^7^ research into the effect of political parties on health and welfare policies is mostly limited to the traditional Social Democratic, Christian Democratic and liberal parties.^12^ This review specifically focuses on the health and welfare policy consequences of PRR participation in government in Europe, thereby adding to our existing understanding of the recent developments in public health.

The aim of this scoping review is to map the available evidence regarding the influence of PRR parties on welfare policy reforms in Europe. Welfare policy was used as a proxy for population health and health equity because of the lack of literature about the direct relationship between PRR parties and health. In comparison, there is an overwhelmingly positive association between welfare policies and health outcomes.^13,14^ Welfare policy centres around a redistribution of economic prosperity across different groups in society through progressive taxation and/or social benefits and provisions.

The emergence of PRR parties plays out differently across different countries in Europe, eg, in access to welfare provisions. Seeking explanations for this phenomenon, the research question of this review is: *How do PRR parties’ ideologies and actions influence welfare policy reforms, and what evidence is available on how this relationship is mediated by characteristics of the political system in different European countries?*

## Methods

In order to collect evidence on the relationship between PRR presence and welfare policies from original research, a scoping review was conducted based on the Methodology for JBI Scoping Reviews by the Joanna Briggs Institute.^15^ A scoping review is a comprehensive literature study with the aim of mapping the available evidence in a particular (emerging) research area. While scoping reviews are similar to systematic reviews, their main goal is to gather insight in key concepts and to assess the extent to which research is available on a topic, rather than to appraise the quality of the available literature.^15^ Given the scarcity of empirical literature about PRR parties and public health, the broader scope and relative flexibility of scoping reviews make it a more appropriate approach.

### Eligibility Criteria

Eligible studies address the influence of *PRR parties* and/or the *political system* on *welfare policy* in Europe. The political system is conceptualised as including the constitution, political economy, interest representation and partisanship.^3^ Welfare policy is used as a proxy for population health and refers to public policies such as healthcare policy, labour market policy and social assistance. Studies assessing associations between any political party category (ie, Christian Democratic, Social Democratic, and liberal parties) and *welfare policies* and/or *population health outcomes* were initially included to give greater insights into the relationship between political ideology and public health in general^16^ (see Supplementary file 1). These studies were excluded in this paper because they could not be directly related to PRR parties. Besides, this review focuses on welfare policy outcomes rather than population health outcomes, as the link between PRR parties and population health outcomes has not yet been studied empirically. This restriction of the eligibility criteria has allowed for a more focused analysis on PRR parties, which allows for new inferences to be made about the pathways through which PRR parties may affect welfare policy. Studies about the influence of the EU on welfare policy were considered because of the important role of the EU in European political systems, especially when it comes to cross-national access to healthcare under EU single market law and EU environmental, occupational and consumer protection policies that indirectly affect health.^17^ In addition, PRR parties are highly critical of the EU.^8^ Studies about the demarcation of the PRR party category and the emergence of PRR parties in European countries were not included. These studies tend to analyse the precedents of PRR parties’ success rather than the possible policy consequences of their participation in government, which is the primary focus of this scoping review. Only original peer-reviewed articles, both qualitative and quantitative, written in English and published after 2000 were eligible. The cut-off date of 2000 was chosen because it coincides with the period in which the majority of PRR parties in (Western) Europe started to participate in national government coalitions (eg, FPÖ and SVP).

### Search Strategy

Relevant studies were identified on the databases PubMed, ScienceDirect and Google Scholar, between February and March 2019. Key words that were used include ‘populist radical right,’ ‘populism,’ ‘political institutions,’ ‘party system,’ ‘welfare state policy’ and ‘health policy,’ used in different combinations (see Supplementary file 1). Additionally, 15 sources were found upon recommendation. Based on the results of the first search, a second search of the same databases has been done with the key words ‘populist radical right AND welfare chauvinism AND European Union.’ This yielded 19 new results on ScienceDirect and 399 on Google Scholar.

### Study Selection

The results of all searches were uploaded to the systematic review web application ‘Rayyan QCRI.’ The study selection process consisted of three stages: title screening, abstract screening and full-text assessment (Figure). After the full selection procedure, 14 articles were identified through the reference lists of eligible articles (‘snowball method’). The full-text assessment was performed twice to ensure the eligibility criteria were met in all included articles. This restricted the sample from 110 articles^15^ after the first full-text assessment to 30 articles, which have been further reduced to 15 articles that were more directly relevant to the research question of this scoping review. As described above, initially included articles addressing political parties other than PRR parties and population health outcomes rather than welfare policy were excluded at this stage.

**Figure:**
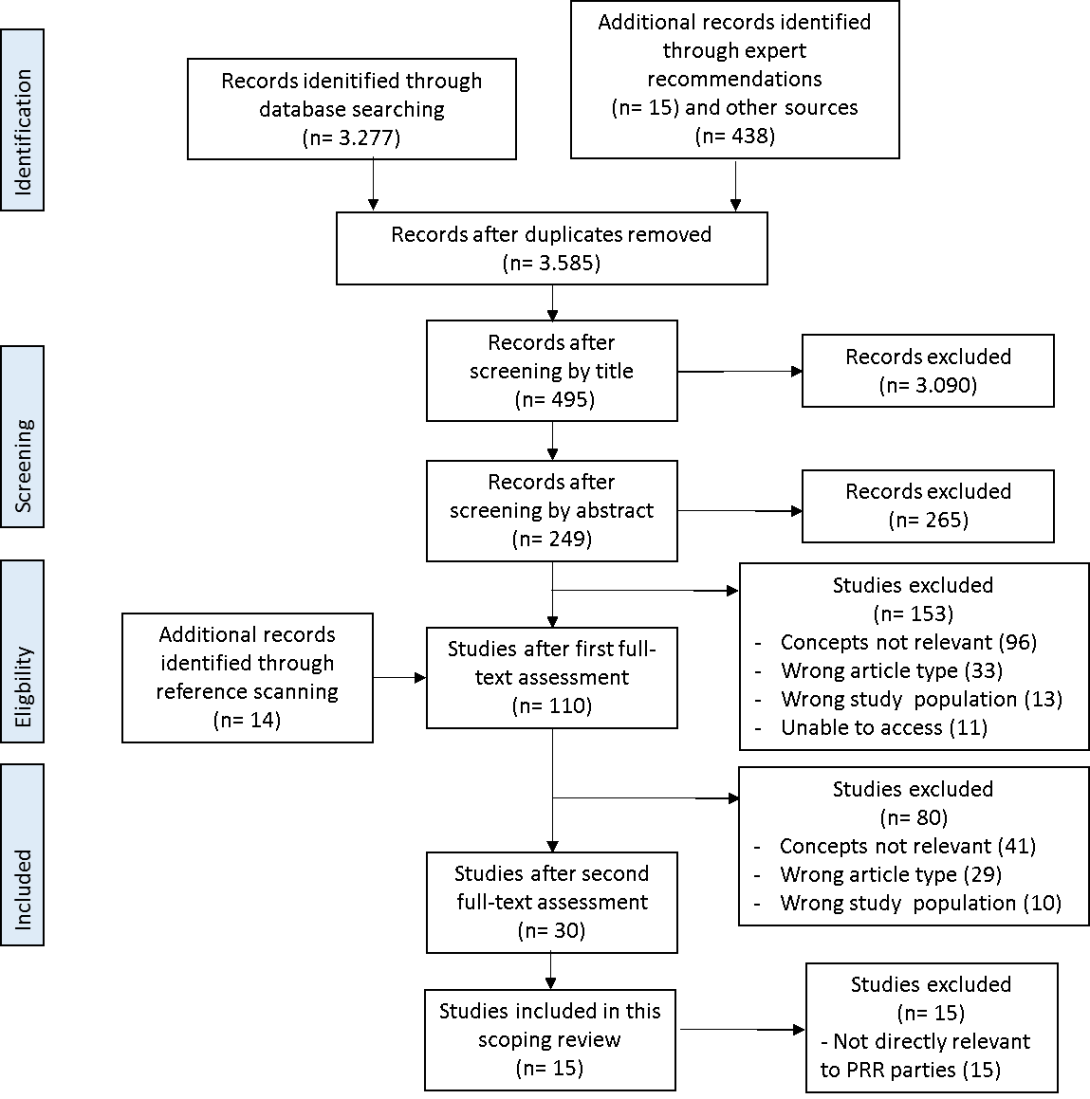
Adapted PRISMA Flow Chart of the Study Selection Process.^14^ Abbreviation: PRISMA, Preferred Reporting Items for Systematic Reviews and Meta-Analyses.

### Data Extraction and Synthesis

The data from the included articles was charted on study characteristics (author, year of publication, journal, title), methods, central concepts (PRR party, political system feature, main outcome measures) and relevant results. Subsequently, a conventional qualitative content analysis was conducted, which is an inductive approach suitable for emerging phenomena.^18^ The data was categorised according to the four political system characteristics: constitution, political economy, interest representation, and partisanship.

### Expert Interviews

Five expert interviews were conducted as validation for the evidence in the literature. Conducting a consultation with experts for qualitative validation is an optional but recommended additional step in the scoping review approach.^15^ Experts are defined as people who have institutionalised expertise in the field of political science through formal training and research and have a publication record on either PRR parties or the politics of healthcare and welfare policies.^19^ Participants were selected based on purposive sampling, using the literature sample of this scoping review and expert recommendations. One of the participants was an author of the reviewed literature.

The interviews were semi-structured and based on the evidence that resulted from the scoping review. Three interviews were conducted face-to-face and two by telephone. All participants received the topic list prior to their interview. By signing an informed consent form, the participants agreed that the interviews would be recorded and transcribed. All participants gave explicit consent for the disclosure of their name and affiliation; however, all information has been handled confidentially. The data was analysed according to the method for analysing expert interviews by Meuser and Nagel.^20^

## Results

Fifteen articles were included in this review (Figure). These articles were published between 2009 and 2018, with nearly three quarters (n = 11) published in 2015 or later. 11 articles directly address the relationship between PRR parties and welfare policy (Table). The most common outcome measures were welfare policy in multiple areas (n = 5) and labour market policy (n = 3). 2 articles discuss the relationship between PRR parties and quality of (liberal) democracy. The political parties that are most represented in the literature sample are the FPÖ, Sweden Democrats (SD), SVP, Dutch Party for Freedom (PVV), Danish People’s Party (DPP) and Italian (Northern) League (LN). These parties are most often referred to as ‘populist radical right parties’ (n = 5) or ‘populist right-wing parties’ (n = 2), ‘far-right populist parties’ (n = 1) and ‘radical right-wing populist parties’ (n = 1), in almost all instances referring to Mudde’s definition of populism, nativism and authoritarianism.^7^ Two articles about the influence of the EU on health(care) policy were also included.

**Table:** Descriptive Summary Table of the Articles About the Relationship Between PRR Parties and Welfare

**Author (Year)**	**Country**	**Years**	**Type of Analysis**	**PRR Parties**	**Main outcome Measures**	**Relevant Results**
Pavolini et al (2018)	Denmark, England, Germany, Italy, Turkey	2011-2018	Mixed methods: quantitative analysis, comparative case study analysis	DPP, UKIP, AfD, LN, JDP	Healthcare policy	The populist discourse around healthcare is stronger in countries with a tax-based healthcare system, low trust in medical professionals, and where major healthcare reforms took place.
Nordensvard and Ketola (2014)	Sweden, Finland	2009-2012	Qualitative policy analysis	SD, PS	Welfare policy in multiple areas	PRR parties reframe the Nordic welfare state model in exclusionary ways (welfare chauvinism) to protect “the people” from threats by Europeanization (PS) and immigration (SD).
Afonso (2015)	Austria, the Netherlands, Switzerland	1995-2012	Qualitative comparative case study analysis	FPÖ, PVV, SVP	Labour market reforms	After facing electoral loss PRR parties prefer supporting welfare policies that benefit their electorate (votes) rather than making compromises with conservative coalition partners (office).
Afonso and Papadopoulos (2015)	Switzerland	1990s-2000s	Mixed methods case study analysis	SVP	Labour market reforms	The SVP formed coalitions with conservative parties on the domains of welfare thought to affect the ‘undeserving,’ but took a milder stance on welfare issues that affect its supporters, such as pensions and healthcare.
Otjes et al (2018)	11 Western European countries	2005-2015	Qualitative comparative case study analysis	FPÖ, VB, DPP, PS, FN, LN, PVV, FrP-N, SD, SVP, UKIP	Welfare policy in multiple areas	European PRR parties show similarities in economic nativism and populism, manifested in welfare chauvinism, while some also show economic authoritarianism.
Careja et al (2016)	Denmark	1975-2011	Mixed methods case study analysis	DPP	Labour market reforms	The participation of DPP in government resulted in limited direct welfare chauvinism (welfare retrenchment for immigrants) and more extensive indirect welfare chauvinism (welfare retrenchment that disproportionally affects immigrants).
Schumacher and van Kersbergen (2016)	Austria, Denmark, Finland, France, the Netherlands, Norway, Sweden	1994-2011	Mixed methods: quantitative cross-country analysis, qualitative comparative case study analysis	FPÖ, BZÖ, FrP-D, DPP, PS, FN, LPF, PVV, FrP-N, SD	Welfare policy in multiple areas	Mainstream conservative parties have been found to accommodate towards PRRs welfare chauvinism, while centre-left parties have responded to the PRR by taking a more critical position against multiculturalism to avoid losing votes to the PRR.
Albertazzi and Mueller (2013)	Austria, Italy, Poland, Switzerland	-	Qualitative comparative case study analysis	FPÖ, LN, PiS, SVP	Quality of democracy	Populist parties challenge liberal democracy eg, by trying to implement anti-minority policies and limiting the power of the juridical system. These policies are often blocked by national and EU judicial institutions.
Ennser-Jedenastik (2017)	The Netherlands, Sweden, Switzerland, UK	2000-2015	Qualitative comparative case study analysis	PVV, SD, SVP, UKIP	Welfare policy in multiple areas	Welfare chauvinism towards healthcare policy was higher in countries with tax-based healthcare systems, compared to insurance-based systems.
Röth et al (2018)	17 Western European counties	1970-2010	Mixed methods; quantitative analysis, qualitative case study	Multiple PRR parties	Labour market reforms	Compared to governments with a liberal ideology, governments with PRR parties showed less political will for welfare retrenchment, likely due to the welfare chauvinistic position of their voters.
Huber and Schimpf (2016)	30 European countries	1990-2012	Quantitative cross-sectional analysis	Multiple PRR parties	Quality of democracy	PRR parties in government decreased democratic quality on average. There is no effect when PRR parties are in opposition and a smaller effect in large coalition governments.
Tyrberg and Dahlström (2017)	Sweden	2015	Quantitative analysis	SD	Aid to vulnerable immigrants	In municipalities where the SD holds a pivotal position and can put direct pressure on its coalition partners, less aid was offered to vulnerable immigrants from the EU.
Ennser-Jedenastik (2016)	Austria	1983-2013	Qualitative case study	FPÖ	Welfare policy in multiple areas	The stance of the FPÖ on social policies is welfare chauvinistic and in line with nativism, authoritarianism and populism since 2005.
Vollaard et al (2013)	EU member states	2003-2012	Qualitative policy analysis	-	Health(care) policy	The scope of EU health(care) policy has expanded, despite healthcare not being an EU competence and members States’ reluctance to EU involvement in healthcare.
Lamping and Steffen (2009)	EU member states	-	Qualitative policy analysis	-	Health(care) policy	The EU has significant, yet fragmented, influence on member states’ health systems. This influence is direct (public health crises), indirect (market integration laws) and politically driven (Europeanization of health expenditure and coverage).

Abbreviations: PRR, populist radical right; FN, National Rally (France); AfD, Alternative for Germany; BZÖ, Alliance for the Future of Austria; DPP, Danish People’s Party; FPÖ, Freedom Party of Austria; FrP-D, Progress Party Denmark; FrP-N, Progress Party Norway; JDP, Justice and Development Party (Turkey); LN, (Northern) League (Italy); LPF, List Pim Fortuyn (Netherlands); PiS, Law and Justice (Poland); PS, True Finns/Finns Party; PVV, Party for Freedom (Netherlands); SD, Sweden Democrats; SVP, Swiss People’s Party; UKIP, United Kingdom Independence Party; VB, Flemish Interest; EU, European Union.

### Content Analysis

This section will start with the empirical evidence on the position of PRR parties on welfare policy, which significantly differs from that of Social Democratic, Christian Democratic, and liberal parties.^10,21^ The extent to which this position, called welfare chauvinism, is applied or becomes effective will be discussed using the four political system characteristics discussed above (constitution, political economy, interest representation and partisanship).

### Welfare Chauvinism and Welfare Policies

A common tendency of PRR parties is to engage in ‘welfare chauvinism.’^10,21-27^ Welfare chauvinism involves increasing welfare provisions for the population belonging to the hypothetical ‘in-group,’ while limiting access and eligibility to welfare provisions for certain vulnerable population groups (mostly immigrants and minorities). According to Ennser-Jedenastik^10^ welfare chauvinistic ideas are an extension of nativism (eg, giving preference to the native population in social security and healthcare) and authoritarianism (eg, exclusive government assistance to those ‘morally deserving’ of support, such as the elderly who have worked and contributed to society). This explanation was also shared among the experts that were interviewed. Due to the question of deservingness, labour market reforms, including pensions and unemployment benefits, were the most studied in relation to welfare chauvinism as these are believed to be especially susceptible to it.^23,24^ One of the experts added that patients are considered one of most deserving population groups by PRR parties, which could make welfare chauvinism in the healthcare policy area especially pronounced. While most articles did not exclusively focus on welfare chauvinism in relation to healthcare, welfare chauvinistic ideas are shared by all PRR parties that were discussed in the literature. Even in the Nordic countries, known for their generous welfare systems, welfare chauvinism is commonly used in PRR electoral manifestos.^24-26^ The Sweden Democrats, for example, called for a restriction of free healthcare services for immigrants.^27^

Once PRR have entered government, welfare chauvinistic standpoints can be translated into policies that either directly exclude the ‘undeserving’ from welfare provisions, or indirectly exclude them through policies that are targeted at the entire population but affect ‘undeserving’ (immigrant) populations disproportionally, such as policies that restrict eligibility to unemployment benefits.^24^ Those in need for healthcare are commonly considered ‘deserving’ of state support (authoritarianism), yet there are indications from multiple PRR parties that this does not apply to citizens belonging to the ‘out-group’ (nativism). Once implemented, welfare chauvinistic policies might therefore have a negative effect on access to welfare provisions for immigrants and minority groups. While indirect welfare chauvinism is more common in most countries in Europe, welfare benefits to immigrants have directly been retrenched under the influence of PRR parties in government in Denmark (social assistance) and Sweden (aid to vulnerable EU immigrants).^24,26^

Similar standpoints thus play out differently across countries once the PRR enters the elected government. We will now investigate possible explanations for these policy differences by focusing on the varying characteristics of political systems across European countries.

### Constitution

The existence of a constitution is an important aspect of liberal democracy, here defined as “a representative government operating through law, by regular, free and fair elections based on universal suffrage, and by respect for individual rights including freedom of expression and association” (p.166).^28^ The PRR tends to have a conflictual relationship with constitutional rights in democracies, which has consequences for the redistribution of benefits in society. This was found both in the literature sample and the expert interviews.

On one hand, (direct) democracy is strongly in line with the populist ambition to be a voice for ‘the people.’ PRR parties have indeed been able to appeal to a part of the electorate that did not feel represented in politics anymore.^29^ Furthermore PRR parties adhere to the ‘rules’ of democracy by participating in elections and using democratic policy-making procedures. One the other hand, constitutional checks and balances, the rule of law and minority rights have been criticized by populist leaders for limiting the extent to which parties can directly translate their positions into policies.^30^ In the last decades, PRR parties have challenged checks and balances through attempts to limit freedom of speech (Poland) and the power of the juridical system (Italy and Poland), open scepticism towards the rule of law (Italy and Austria) and the implementation of anti-immigrant and anti-minority policies, including those restricting welfare benefits. Such challenges are suggested to stem from the combination of the populist desire for direct democracy and the nativist and authoritarian elements of the PRR ideology. Indeed, PRR participation in government has been associated with decreased democratic quality on average.^29^

But what do we know about the influence of constitutional rights and order on the adoption of the PRR agenda? Evidence from the included literature suggests that judicial institutions have a mediating role.

#### Judicial Institutions

Considering the conflictual relationship between the PRR and liberal democracy, enforcement of the constitution through juridical review has had a constraining effect on the political agenda of these parties.^10,24,30^ Examples are the blocked proposals by the FPÖ (Austria) to implement contributions for patients that make excessive use of medical treatment (indirect welfare chauvinism)^10^ and their directive to withdraw any form of state support for asylum seekers (direct welfare chauvinism).^30^ However, some countries, like the United Kingdom, do not have a Constitutional Court or even a Constitution to which public policies can be reviewed. In EU member states, illiberal policies are also reviewed against EU law and conventions by supranational institutions. In Italy, PRR policy proposals have been blocked by European institutions (European Court of Human Rights and European Court of Justice) because of violation of EU laws and conventions. These policy proposals most often targeted the restriction of checks and balances or immigration law.^30^ However, restrictions of state support to immigrants translates to cuts in welfare benefits, such as social assistance and access to healthcare. In this light, judicial institutions such as EU institutions and constitutional courts to some extent restrict direct welfare chauvinism and challenges to democracy. This idea was supported by three of the interviewed experts, on the condition that welfare chauvinistic policies directly discriminate based on origin/citizenship. The two other experts believed the link between judicial institutions at national and EU level and PRR parties’ welfare policies to be too indirect. However, one of them highlighted that restriction of access to welfare (ie, welfare chauvinism) can be considered a direct violation of the ‘social rights of democracy.’

### Political Economy

The political economy includes trade policies, fiscal policies, labour market policies and socio-economic policies. While variation exists, PRR parties’ standpoints on economic interventionism were found to correspond to those of mainstream centre-right parties (ie, favouring financial and labour market deregulation).^21,31^ However, no clear connection was found between economic interventionism and the PRR ideology based on populism, nativism and authoritarianism.^25^ When it comes to redistributive socio-economic policies, such as pensions, social assistance and access to healthcare, the extent to which PRR parties emphasize welfare chauvinism possibly depends on how the welfare programme is funded.^22,27^

#### Tax-Based Healthcare Systems

Some PRR parties de-emphasize health in favour of immigration and security policies^32^ while others include specific claims about healthcare in their party manifestos. There is evidence that the extent to which welfare chauvinism is directed towards the healthcare system differs between countries with tax-based and insurance-based healthcare systems.^27^ Tax-funded universal systems based on principles of equality are more in contrast with the PRR’s nativist beliefs and therefore expected to be more susceptible to welfare chauvinistic appeals.^27^ Indeed, welfare chauvinistic claims targeted at the healthcare system were found to be more frequent in the United Kingdom, Sweden and Italy, which have tax-based healthcare systems, compared to the Netherlands, Switzerland, and Germany, where healthcare is organised based on a mandatory private insurance scheme.^22,27^ In countries with an insurance-based system, exclusion from healthcare was only emphasized for those who did not yet financially contribute to the healthcare system. Similar patterns exist regarding pensions, unemployment and social assistance, yet welfare chauvinism in those areas was found to be weaker.^27^ In addition, populist discourse around healthcare seems to be more prominent in countries who introduced cost-cutting healthcare reforms and where trust in the healthcare system is lower.^22^

There is thus evidence that PRR welfare chauvinistic standpoints vary according to healthcare system related variables in different countries, yet no evidence was found about differences in implementation of welfare chauvinistic policy reforms in countries with different types of healthcare systems. The experts with whom this hypothesis was discussed more in-depth believed it to be plausible but highlighted that it is an ‘open’ hypothesis that requires further research.

#### European Single Market

In the political economy of EU member states, the European single market regulations play a significant role in trade and labour market policies. The EU internal market, which is a form of ‘hard’ EU law, unites the member states’ markets into one single market where citizens have the right to use and provide (healthcare) services across member states.^33^ Because all member states need to abide to EU law, the EU internal market law limits the extent to which national governments can arrange health and welfare provisions, and acts against national policies that constrain free movement of citizens and healthcare services,^33,34^ as proposed by PRR parties in the United Kingdom^22,27^ and Finland.^25^ One of the expert participants called the EU single market a ‘restraint’ for the PRR policy agenda, while another expert related this directly to the Euroscepticism that is often associated with PRR parties.

### Interest Representation

No clear evidence regarding the possible mediation of interest representation in the relationship between welfare chauvinism and welfare policies was found in the literature sample of this scoping review. However, two experts mentioned that a corporatist political system that is more consensual in nature, such as in the Netherlands, could restrain ‘extreme’ policy-making as it ‘forces’ parties to engage and form a compromise. The possible role of interest representation in PRR welfare policy reforms is further discussed in the discussion paragraph.

### Partisanship

The possible mediating role of partisanship focuses on the extent to which PRR parties and mainstream parties respond to electoral preferences and engage in vote-seeking and office-seeking behaviour. Most PRR parties in Europe have entered the executive government office in centre-right government coalitions with ambitions for welfare retrenchment.^23^ PRR parties thus face a trade-off between supporting the retrenchment proposals of their coalition partners to establish a coalition agreement (office-seeking behaviour) or enforcing welfare policies that benefit their electorate more directly (vote-seeking behaviour).^23,31,32^ Based on case studies from Austria, Switzerland and the Netherlands, Afonso^23^ suggests that the extent to which PRR parties support the pension retrenchment initiatives by their coalition partners depends on the likelihood of electoral losses. Whenever PRR parties in office faced electoral losses following the introduction of welfare retrenching policies, they tended to prioritise their voters’ direct preferences, leading to increased welfare chauvinistic positions. In the Netherlands, vote-seeking strategies by the PRR eventually led to the fall of government. In Denmark, on the other hand, the DPP successfully combined vote- and office-seeking strategies, thereby claiming credit for exclusionary labour market measures (eg, residence criteria for pensions), while at the same time avoiding electoral blame for welfare retrenchment and retaining their position in office.^24^ All experts agreed that vote-seeking behaviour could increase welfare chauvinism. However, whether PRR parties choose this strategy over office-seeking behaviour was thought to depend on the compromises they were able to make with their coalition partners. One expert mentioned that PRR parties might be more willing to make compromises on healthcare policy than on immigration policy.

#### Accommodation of Welfare Chauvinism by Mainstream Parties

Evidence also shows how the strategies of PRR parties in government influence the positions of mainstream parties.^24,26,35^ Schumacher and van Kersbergen found that, as PRR parties shifted towards a more welfare chauvinistic position, conservative parties accommodated these positions in order to increase their electoral share at the expense of the PRR.^35^ While Social Democratic parties did not change their position on welfare, they did become more sceptical of multiculturalism, especially after facing electoral losses. Accommodation by mainstream parties might also occur once in office when PRR support is needed to establish a majority government, leading to the actual implementation of welfare chauvinistic policy reforms.^24,26^ For example, less aid was given to vulnerable immigrants from the EU in Swedish municipalities where the SD had more bargaining power due to its pivotal position.^26^ In Denmark, the access to benefits for immigrants was restricted with PRR support.^35^

In summary, partisanship does mediate the influence of PRR ideologies on welfare policies in different ways. PRR parties that initially compromised on welfare issues with their right-wing coalition partners (office-seeking behaviour) have altered their position following electoral losses to match the nativist preferences of their electorate (vote-seeking behaviour). Mainstream parties have been found to accommodate welfare chauvinism both for electoral reasons (vote-seeking behaviour) or when in need of a government majority (office-seeking behaviour), which has at least in two known cases (Sweden and Denmark) resulted in (local) welfare chauvinistic policies.

## Discussion

This scoping review analysed empirical evidence of PRR party influence on welfare policy reforms and the possible implications for population health. This section will continue with a discussion of the implications of welfare chauvinism and the possible role of political system characteristics as mediator between the PRR political agenda and the implementation of exclusionary welfare reforms.

### Welfare Chauvinism

Welfare chauvinism is the most prominent channel through which PRR parties could adversely affect population health and health equity in Europe, as welfare chauvinistic policies have the potential to directly affect access to welfare provisions for vulnerable (immigrant) groups. Both universal access to healthcare and other redistributive welfare provisions, such as pensions and unemployment benefits, have been associated with increased population health either directly or indirectly.^13,14^ This confirms the idea that PRR parties could pose a threat to population health due to their exclusionary policy agenda,^3,6,9^ especially since positive effects of welfare chauvinism for the native population are not clear.^36^ While direct welfare chauvinism is more likely to make immigrant and minority groups especially vulnerable, indirect welfare chauvinism might affect a bigger proportion of the population, including the native ‘in-group.’ Welfare chauvinism might thus represent a paradox in which it harms its very own proponents, especially the most vulnerable (eg, people who are unemployed).

### Mediation by Political System Characteristics

The influence of the PRR on welfare policy, and possibly population health, is hypothesized to be mediated by the characteristics of political systems in different countries. Except for interest representation, on which no clear evidence was found, all components of the political system appear to play a mediating role.

The political economy explains differences in the welfare chauvinistic standpoints of PRR parties in different countries. Tax-funded healthcare systems appear to be more exposed to welfare chauvinistic claims than insurance-based systems. Besides the difference in redistributive principles behind healthcare systems – with tax-based systems based on the principle of equality being most at odds with the PRR ideology^27^ - PRR parties associate government-led healthcare systems with the ‘corrupt elite.’ Insurance-based systems are privatised and thus less susceptible to public scapegoating. Nevertheless, PRR parties in countries with insurance-based systems might use a ‘softer’ form of exclusion or re-direct welfare chauvinistic appeals at specific tax-based healthcare components, as the PVV did with long-term care for the elderly in the Netherlands.

When it comes to the implementation of welfare chauvinistic policy reforms, partisanship likely plays a mediating role. As welfare chauvinism becomes more mainstream among the electorate in European countries, both PRR parties and traditional parties have an incentive to support welfare chauvinistic policies. The cases in this review are based on corporatist systems with coalition governments where PRR parties are not the biggest party (eg, Switzerland, Austria, Denmark, the Netherlands). In such corporatist systems of interest representation, electoral preferences are represented in multiparty systems that traditionally prevent single party dominance, necessitating coalition governments based on a coalition agreement. The result is a relatively stable long-term policy pathway towards societal development. Pluralist systems on the other hand consist of polarised two-party systems with adversarial relationships and short-term focus. Generally, coalition agreements are argued to form a buffer against the implementation of exclusionary welfare reforms, whereas majority governments are more likely to implement polarised or ‘extreme’ policies.^36^

While no empirical evidence was found about the influence of interest representation on welfare chauvinism, mainstream parties of either side of the political spectrum were found to allow or even propose exclusionary welfare policies as a reaction to PRR electoral success (eg, in Denmark, Sweden, and Austria).^35^ Based on these findings, the presumed protective role of coalition governments against the PRR’s welfare chauvinistic policy agenda, seems limited. However, accommodation has also been argued to be a short-term strategy used by mainstream parties to ‘disarm’ PRR parties.^42^

An additional, less established, channel through which PRR parties could affect population health is through their challenges to democratic principles and the EU. Countries with a liberal democratic political system show more positive results on populations health indicators on average. This is most likely due to improvements in socio-economic factors (eg, income, education and social access to healthcare).^28,37^ Furthermore, political oppression of certain population groups and the infringement of human rights could lead to negative psychosocial experiences that can affect citizens’ mental and physical health.^37^ In this light, the erosion of the constitution (rule of law, separation of powers and minority rights) by PRR parties in office could theoretically have negative consequences for public health (eg, causing negative psychosocial experiences). This relationship is yet to be studied empirically.

While no direct health benefit of EU membership was found,^38^ the EU has considerable influence on national health(care) policy through cross-border security measures, trade of medicines and healthcare services (European single market)^33^ and environmental, occupational and consumer protection policies.^17^ Not surprisingly, the EU has a bad reputation among PRR party leaders and voters for being ‘elitist’ and threatening national sovereignty.^39^ The clearest example of this is Brexit, in which the rejection of free movement of EU citizens and their right to use the National Health Service played a central role.^22^ Besides, PRR parties and their supporters believe that increased (economic) migration of EU citizens to Western European countries is one of the main reasons for the worsened economic position of the native working class. Withdrawal from the EU, which forms a central agenda point for several PRR parties, could have serious implications for public health. Indeed, Brexit is forecasted to have negative consequences for among others healthcare financing, healthcare workforce, access to medicines, blood and tissues, and health research.^40,41^

With PRR parties that continue to gain power as they prioritise their voters’ populist and nativist beliefs and mainstream parties that are willing to accommodate welfare chauvinistic positions, constitutions and EU laws might become increasingly important in safeguarding democratic principles and protecting vulnerable minorities such as ‘illegal’ immigrants and asylum seekers. When it comes to access to healthcare in specific, the European single market forms an extra layer of protection.

### Strengths and Limitations

To date, this is the first scoping review that analyses original empirical research about the relationship between PRR parties in Europe and health and welfare policy, investigating the possible mediating role of political systems in PRR parties’ welfare policy influence. To ensure the scientific quality of the analysed evidence, strict eligibility criteria were set for the data and a consultation with experts was conducted for validation purposes. However, this also means that a limited literature sample was used. Like in any literature review, the conclusions in this review are dependent on the availability of literature, which made it necessary to use a proxy for population health. Besides, the views of experts and authors cannot be free from bias. Expert respondents were partly selected based on the literature sample of the scoping review, meaning that there could have been a selection bias towards a certain perspective due to personal political beliefs and preferences. As for our own position, we have taken up this research out of concern with recent public health trends and have refrained from interpretation if the evidence was too weak.

### Implications for Research and Practice

The popularity of the welfare chauvinistic PRR ideology and its possible effects on population health emphasise the need for empirical research assessing the public health consequences of PRR parties in office. Future research is warranted to investigate the types and dynamics of interest representation, comparing corporatist and pluralist systems, as additional explanations for variations in PRR welfare policy influence.

Recent developments offer new insights, as PRR parties have now also entered coalition governments as the biggest party, for example in Hungary, Turkey and Poland. In Poland, not immigration policy, but welfare chauvinism is central to the policy agenda of PRR party Law and Justice.^43^ Indeed, the party was re-elected as the biggest party in the 2019 national elections due to its investments in the Polish welfare state. However, these are relatively turbulent political times. In Denmark, for example, the Social Democrats have been elected after the previous conservative coalition government including the DPP, adopting parts of its nativist agenda.

This analysis finds both protective and risk factors in political systems that influence PRR parties’ effects on welfare policy, and consequently public health. It is of importance for the public health community to create awareness for harmful PRR beliefs and policies and join forces with other public values. Using a systems perspective that includes political variables is increasingly necessary in monitoring population health developments and evaluating health(care) policies.

## Conclusion

Early evidence from original research indicates that PRR parties influence welfare policy through their welfare chauvinistic agenda aiming for the restriction of access and eligibility to welfare provisions for certain vulnerable immigrant and minority groups. Considering the well evidenced positive relationship between welfare generosity and population health outcomes, it is likely that welfare chauvinism has negative implications for health equity and population health. Accommodation to welfare chauvinism by mainstream parties and recent political developments in Europe, such as the (re)election of PRR parties with an absolute majority in Hungary and Poland, and Brexit, indicate the increasing influence of the PRR ideology on welfare policy. At the same time, political system characteristics in different European countries, such as constitutional regulations and the political economy, appear to limit the extent to which the welfare chauvinistic policy agenda is translated into policy reforms, therefore weakening the possible negative effects on society.

## Acknowledgenments

An earlier version of this review has been presented during the 12th European Public Health Conference held in November 2019. The conference abstract is available at https://doi.org/10.1093/eurpub/ckz185.782. No funding was received for this study.

## Ethical issues

Not applicable.

## Competing interests

Authors declare that they have no competing interests.

## Authors’ contributions

CR drafted the paper. MPMB provided supervision and revised the paper.

## Supplementary file

Supplementary file 1. Search Strategy and Article Inclusion.
