# Supplementary material for: A Scoping Review of Populist Radical Right Parties’ Influence on Welfare Policy and its Implications for Population Health in Europe
Source: Int J Health Policy Manag. 2020 Apr 8;10(3):141–51. doi: 10.34172/ijhpm.2020.48 (PMC7947904; doi:10.34172/ijhpm.2020.48)
Supplement: Supplementary file 1 — Search Strategy and Article Inclusion. [file ijhpm-10-141-Supp1.pdf]

Supplementary file 1. Search Strategy and Article Inclusion

Full search strategies in the databases PubMed, ScienceDirect and Google Scholar.

**Database**	**Search strategy**	**Results**
PubMed	((populism OR "populist radical right") OR ("political system" OR democracy OR "political institutions" OR "veto points" OR "political parties" OR "party system")) AND (“health policy” OR "healthcare policy” OR "welfare state policy" OR "public health")	955
ScienceDirect	((populism OR "populist radical right") OR ("political system" OR "democracy" OR "political institutions" OR "veto points" OR "political parties" OR "party system")) AND (“health policy” OR "healthcare policy" OR "welfare policy" OR "public health")	1.352
Google Scholar	((populism OR "populist radical right") AND ("political system" OR "political parties")) AND ("health policy" OR "welfare state policy")	970

Reference list of included articles

1. Afonso A, Papadopoulos Y. How the populist radical right transformed Swiss welfare politics: from compromises to polarization. *Swiss Political Sci Rev*. 2015;21(4):617-635. doi: 10.1111/spsr.12182
2. Afonso A. Choosing whom to betray: populist right-wing parties, welfare state reforms and the trade-off between office and votes. *Eur Political Sci Rev*. 2015;7(2):271-292. doi: 10.1017/S1755773914000125
3. Albertazzi D, Mueller S. Populism and liberal democracy: Populists in government in Austria, Italy, Poland and Switzerland. *Gov Oppos*. 2013;48(3):343-371. doi: 10.1017/gov.2013.12
4. Careja R, Elmelund-Præstekær C, Baggesen Klitgaard M, Larsen EG. Direct and indirect welfare chauvinism as party strategies: an analysis of the Danish People's Party. *Scand Political Stud*. 2016;39(4):435-457. doi: 10.1111/1467-9477.12075

5. Enner-Jedenastik L. A Welfare State for Whom? A Group-based Account of the Austrian Freedom Party's Social Policy Profile. *Swiss Political Sci Rev*. 2016;22(3):409-427. doi: 10.1111/spsr.12218
6. Ennser-Jedenastik L. Welfare chauvinism in populist radical right platforms: The role of redistributive justice principles. *Soc Policy Adm.* 2018;52(1):293-314. doi: 10.1111/spol.12325
7. Huber RA, Schimpf CH. A drunken guest in Europe?. *Zeitschrift für vergleichende Politikwissenschaft.* 2016;10(2):103-129. doi: 10.1007/s12286-016-0302-0
8. Lamping W, Steffen M. European union and health policy: the “chaordic” dynamics of integration. *Soc Sci Q.* 2009;90(5):1361-1379. doi: 10.1111/j.1540-6237.2009.00659.x
9. Nordensvard J, Ketola M. Nationalist Reframing of the Finnish and Swedish Welfare States– The Nexus of Nationalism and Social Policy in Far-right Populist Parties. *Soc Policy Adm*. 2015;49(3):356-375. doi: 10.1111/spol.12095
10. Otjes S, Ivaldi G, Jupskås AR, Mazzoleni O. It's not Economic Interventionism, Stupid! Reassessing the Political Economy of Radical Right-wing Populist Parties. *Swiss Political Sci Rev*. 2018;24(3):270-290. doi: 10.1111/spsr.12302
11. Pavolini E, Kuhlmann E, Agartan TI, Burau V, Mannion R, Speed E. Healthcare governance, professions and populism: Is there a relationship? An explorative comparison of five European countries. *Health Policy*. 2018;122(10):1140-1148. doi: 10.1016/j.healthpol.2018.08.020
12. Röth L, Afonso A, Spies DC. The impact of Populist Radical Right Parties on socio-economic policies. *Eur Political Sci Rev*. 2018;10(3):325-350. doi: 10.1017/S1755773917000133
13. Schumacher G, Van Kersbergen K. Do mainstream parties adapt to the welfare chauvinism of populist parties?. *Party Politics*. 2016;22(3):300-312. doi: 10.1177/1354068814549345
14. Tyrberg M, Dahlström C. Policy effects of anti-immigrant party representation on aid to vulnerable European union/European economic area citizens. *Political Stud.* 2018;66(1):3-22. doi: 10.1177/0032321717722361
15. Vollaard H, van de Bovenkamp HM, Vrangbæk K. The emerging EU quality of care policy: From sharing information to enforcement. *Health Policy*. 2013;111(3):226-233. doi: 10.1016/j.healthpol.2013.05.004

Articles excluded from this scoping review

The descriptive summary table below presents articles about the relationship between various features of the political system and welfare policy or population health outcomes. While these studies were included in the original analysis that was performed, the evidence could not be directly related to PRR parties and their welfare policies and was therefore excluded from the sample for this paper. A portion of these studies was considered in the Discussion section on this paper.

**Author (Year)**	**Country**	**Years**	**Explanatory Political Variables**	**Main Outcome Measures**	**Relevant Results**
Regidor, Pascual, Martinez, Calle, Ortega, & Astasio (2011)	17 Western countries (15 European)	1900-2005	Political ideology, family policy regime	Infant mortality rate	Countries with a socio-democratic political tradition and countries with a Scandinavian model of family policy had the lowest rates of infant mortality. However, this was likely a consequence of policies that were independent from political ideology.
Navarro, & Shi (2001)	OECD countries	1945-1980	Political ideology	Infant mortality rate	Strong trade unions and socio-democratic governments were found to be more committed to redistribution than Christian Democratic and liberal Anglo-Saxon governments, and therefore had lower infant mortality rates and social inequalities.
Mackenbach, Hu, & Looman (2013)	29 European countries (incl. Soviet Union)<br>43 European	1960-1990<br>1987-2008	Level of democracy, cumulative years of democracy	Life expectancy, adult mortality rate	During 1960-1990 a positive association was found between current democracy level and life expectancy. In the time-period 1987-2008 a higher life expectancy was associated with cumulative years of democracy. The observed

	countries (incl. newly independent republics)				increase in life expectancy was mediated by changes in health outcomes, and likely by modernization of the healthcare sector and economic restructuring.
Navarro, Muntaner, Borrell, Benach, Quiroga, Rodríguez-Sanz, Vergés, Pararín (2006)	OECD countries	1950–2000	Power resources, labour market variables, welfare state variables, economic inequality	Infant mortality rate, life expectancy	A positive association was found between social democratic political tradition and redistributive policies and population health outcomes. It is suggested that political ideology affects population health outcomes through the mediation of welfare and labour market policies and economic inequality.
Mackenbach & Looman (2013)	25 European countries	1955-1989	Level of democracy	Adult mortality rate	The association between mortality rate and democracy became more negative over time. The difference in mortality between Western and Central/Eastern European countries could be explained by differences in democracy, as democratic governments are thought to be more engaged in health promotion than authoritarian governments.
Safaei (2006)	118 countries	2002, 2003, 2005	Level of democracy, socio-economic factors	Life expectancy, child mortality rate, adult mortality rate	Democracy was found to have a direct effect on population health measures. However, socio-economic factors such as income and social access to healthcare services partly mediate this relationship.
Chung, & Muntaner (2007)	OECD countries	1960-1998	Welfare regime	Infant mortality rate	Population health status differs by welfare regime type. Social democratic countries show the best population health status in

					the period 1960-1998, compared to Christian Democratic, liberal and wage earner countries.
Mackenbach (2013)	46 European countries (including former USSR)	1900-2008	Level of democracy, EU membership	Life expectancy, adult mortality rate	Western countries with a long history of democracy had better population health than former USSR and Yugoslavian countries. Differences between democratic and authoritarian countries varied in the period of study, influenced by (civil) wars, oppression and investment in public health interventions. EU membership was not found to have converging effects on life expectancy.
Chung, & Muntaner (2006)	OECD countries	1960-1994	Political variables, welfare state variables, Gini coefficient	Infant and child mortality rate	An association was found between the provision of public medical services and child health outcomes, while Gini coefficient, voter turnout and left vote were weak predictors. The influence of left vote diminished after welfare policies were included in the model, suggesting that a social democratic government has influence on population health through pro-welfare policies.
Mackenbach, & McKee (2013)	43 European countries	ca. 2000	Level of democracy, political composition of government, government effectiveness	Implementation of preventative health policy, impact of health policies on exposure to health risks, health impacts	Health policy performance was mostly found to be predicted by self-expression values and ethnic fractionalization. However, in specific policy domains, national income and government effectiveness were also predicting of policy performance. Left-wing parties

				of health policies	seem to have little effect on the implementation of preventative health policies.
Reeves, McKee, Basu, & Stuckler (2013)	27 European countries	1995-2011	Political ideology, IMF program participation, GDP change	Public health expenditure	Health care spending was not associated with political ideology of the governing party. Countries that introduced austerity measures, especially IMF/EU lending program participants, significantly cut on healthcare. Austerity policies were more prominent in countries with a tax-financed healthcare system compared to social insurance systems.
Mackenbach, & McKee (2015)	30 European countries	1990-2010	Level of democracy, political representation. distribution of power, quality of government	Implementation of health policy, frequency of behaviours targeted by health policy, frequency of health outcomes of health policy	Only level of democracy was found to be consistently associated with health policy indicators. Distribution of power and political representation were not associated with health policy, despite being associated with other areas of public policy in other studies.
Walker, Anonson, & Szafron (2015)	164 countries (Europe and OECD coded separately)	2011	Level of democracy, functioning of government, political culture, electoral processes, political participation, civil liberties	Health service accessibility	Democracy and health service accessibility are only weakly associated, however the interaction between the sub-components functioning of government and political participation has a positive relationship with health service accessibility.

Tenbensel, Eagle, & Ahston (2012)	11 high income countries (of which 7 Western European countries)	2003-2010	Health care funding type, party composition of government	Health policy objectives	Political factors were not found to have major influence on health policy objectives. However, center-left governments were more likely to put population health outcomes and reduction of health inequalities on their public health agendas, especially in tax-funded health systems. Social insurance systems focused more on efficiency and cost-containment goals.
